# A Fast Multimodal Ectopic Beat Detection Method Applied for Blood Pressure Estimation Based on Pulse Wave Velocity Measurements in Wearable Sensors

**DOI:** 10.3390/s17010158

**Published:** 2017-01-14

**Authors:** Maik Pflugradt, Kai Geissdoerfer, Matthias Goernig, Reinhold Orglmeister

**Affiliations:** 1 Chair of Electronics and Medical Signal Processing, TU Berlin, 10623 Berlin, Germany; Maik.Pflugradt@googlemail.com (M.P.); Kai.Geissdoerfer@mailbox.tu-berlin.de (K.G.); 2 Staedtisches Klinikum Dresden-Neustadt, 01159 Dresden, Germany; Matthias.Goernig@khdn.de

**Keywords:** ectopic beat detection, pulse wave velocity, blood pressure estimation, pulse arrival time, multimodal signal processing, wearable sensor network

## Abstract

Automatic detection of ectopic beats has become a thoroughly researched topic, with literature providing manifold proposals typically incorporating morphological analysis of the electrocardiogram (ECG). Although being well understood, its utilization is often neglected, especially in practical monitoring situations like online evaluation of signals acquired in wearable sensors. Continuous blood pressure estimation based on pulse wave velocity considerations is a prominent example, which depends on careful fiducial point extraction and is therefore seriously affected during periods of increased occurring extrasystoles. In the scope of this work, a novel ectopic beat discriminator with low computational complexity has been developed, which takes advantage of multimodal features derived from ECG and pulse wave relating measurements, thereby providing additional information on the underlying cardiac activity. Moreover, the blood pressure estimations’ vulnerability towards ectopic beats is closely examined on records drawn from the Physionet database as well as signals recorded in a small field study conducted in a geriatric facility for the elderly. It turns out that a reliable extrasystole identification is essential to unsupervised blood pressure estimation, having a significant impact on the overall accuracy. The proposed method further convinces by its applicability to battery driven hardware systems with limited processing power and is a favorable choice when access to multimodal signal features is given anyway.

## 1. Introduction

Ectopic means out of place and originates from the word *ektópios* in the ancient Greek language. Ectopic beats (EB) are heartbeats that are not caused by a normal sinus node pace, but by an electrical potential somewhere else, referred to as the *ectopic focus*. The physiological backgrounds of ectopic beats have been subject to decades of research with accepted understandings and detailed definitions that can be found in basic ECG literature [1].

According to the origin of the ectopic focus, one generally distinguishes between supraventricular ectopic beats (SVEBs) and ventricular ectopic beats (VEBs). SVEBs have their origin above the atrioventricular node, leading to a distorted P wave but an otherwise morphologically normal QRS complex, as the electromechanical propagation occurs through the regular system. VEBs, on the other hand, may arise from any spot within the cardiac muscle and spread through the myocardium in an abnormal manner. The most obvious observations associated with VEBs are a missing P wave, an early, malformed QRS complex and a compensatory pause as a result of the refractory state of the chambers after the ectopic beat [1]. The modified QRS complex is often broader and yields a higher amplitude and energy.

Whereas strobes of SVEBs and, especially VEBs, have a perceptible impact on the cardiac output and can develop into life threatening tachycardias, sporadic occurring extrasystoles usually do not play a significant role and can be considered as a harmless myocardial event that is also experienced in the healthy subject [2]. With respect to automatic signal processing methods on the other hand, even single ectopic beats can indeed have a crucial influence that needs to be accounted for, especially when tight heart rate timings are involved. This is not only a serious issue in heart rate variability (HRV) analysis, but also in further applications, depending on reliable fiducial point detection including pulse transit time (PTT) and pulse arrival time (PAT) investigations.

One very prominent field of application, which is, in turn, directly affected, involves various approaches aiming to solve the problem of continuous blood pressure estimation (BPE). Until today, cuff dependent measurements based on the auscultatory and oscillometric measurement principles are still considered the gold standard for non-invasive blood pressure determination. The systolic blood pressure (SBP) and diastolic blood pressure (DBP) are typically derived by evaluating Korotkoff’s sounds, whereas the mean blood pressure (MBP) is easier to detect and a more reliable surrogate when compared to intraarterial derived measurements [3]. With respect to the intraarterial blood pressure signal, MPB represents the area under the curve and is associated with cardiac output, systemic vascular resistance and mean systemic filling pressure. SBP, on the other hand, is physiologically determined by stroke volume and arterial compliance, whereas DBP is closely connected to the vascular tone [4]. BPE approaches based on pulse wave velocity (PWV) considerations have drawn increasing attention in the last years, as they provide a continuous and non-invasive measurement alternative, which can offer new diagnostic opportunities, especially in ambulatory settings where PAT or PTT measurements are commonly used as a PWV surrogates. A very interesting survey on that topic was recently published by Buxi et al. [5], who depict the basic underlying mechanisms, list current approaches and achievements and also critically highlight open aspects and unsolved issues. The issue of ectopic beats is underlined as an unaddressed problem in PAT/PTT detection, which has motivated the efforts of this work and will be the focus of the following evaluation. This eventually includes a detailed investigation focused on the influence of occurring ectopic beats on the BPE process itself. For this purpose, three different blood pressure estimation models from literature are implemented and analyzed with respect to their classification performance during ectopic beats.

Finally, the utilization of a novel multimodal ectopic beat detector, in order to support unsupervised BPE methods, is advocated, accompanied by a quantitative evaluation of the BPE performance before and after extrasystole cancellation.

The paper is organized as follows: Section 2 starts with a brief review on frequently cited EB detection methods. Section 3 then concerns itself with the influence of ectopic beats on BPE applications. First, a short introduction to non-invasive pulse wave velocity acquisition is given in Section 3.1, followed by a presentation of a wireless hardware system in Section 3.2, which was applied for PWV measurements conducted in this work. Section 3.3 then presents a novel multimodal ectopic beat discrimination algorithm, which aims to support BPE applications that are subsequently introduced in Section 3.4. Next to the conducted experiments, the algorithms have additionally been tested on records drawn from publicly available databases, which are depicted in Section 3.5. Section 4 then provides the classification results of the proposed ectopic beat detector along with a detailed evaluation on ectopic beat clearance in the aforementioned BPE applications. The most important results are summarized and interpreted in Section 5, whereas Section 6 finishes with the drawn conclusions and a short outlook.

## 2. Previous Work

The general problem of heartbeat classification has been a target for research for several decades. This section provides an overview of the most influential approaches and identifies common elements.

Early approaches based on ECG morphology applied the underlying pattern recognition techniques directly on the sampled datapoints around the delineated QRS complexes. This strategy yields good results in distinguishing beats of different morphology, as is the case in normal heart beats and VEBs. In 1992, Chow et al. proposed a method for detecting VEBs in two-lead ECG recordings, which is based on this direct sampling method in combination with a backpropagation artificial neural network (ANN) for the classification task [6]. When trained on data from the same patient, they achieved a sensitivity of 97.39% and a positive predictivity of 93.58%.

Clifford et al. further elaborated upon this idea around 2000 and published relating approaches, where the focus was set on learning patient-specific ECG morphology with auto-associative neural networks [7,8].

A very comprehensive work was proposed by Martis et al. [9] in 2013. The authors, just like their predecessors, cut a window of fixed size around every R-Peak. The single extracts are then filtered using a wavelet analysis. To reduce the dimensionality of the feature space, they compare methods based on principal component analysis (PCA), independent component analysis (ICA) and linear discrimination analysis (LDA). Thereafter, the reduced feature vectors were classified by support vector machines (SVM), ANN and probabilistic neural networks (PNN). The best performance was achieved with the combination of ICA and PNN. The results for specificity, sensitivity, accuracy and precision are all above 99%, tested on the MIT-BIH database (Massachusetts Institute of Technology - Beth Israel Hospital), but the underlying random 10-fold evaluation scheme does not consider generalisation across patients.

More recent methods usually rely on a combination of features including signal amplitudes and time intervals, statistical measures and more abstract features. The work from Chazal et al. [10] introduces a set of morphological features, which were later used and extended by many other researchers. The noise reduced ECG signal is first delineated to determine relevant fiducial points including on- and offsets of QRS-, T-, and P-waves of each heartbeat. Next, the feature extraction stage calculates intervals and amplitudes at different points of the heartbeats. These features are then processed by two LDA classifiers, whose outputs are combined to get the resulting membership function. The reported accuracy of 84.5% on the MIT-BIH database is competitive, as they used data of different patients for training and testing the algorithm.

Some of these features are taken up by Sadiq and Khan in 2011 [11]. Resorting to only nine morphology and interval features, they calculate membership functions and classify the heartbeats with a neuro fuzzy ANN. The reported results of 95% specificity and an average sensitivity of 91.17% for SVEB and VEB beats on the MIT-BIH database are quite impressive, as they did not evaluate their classifier on the data of known patients.

Whereas the majority of ectopic beat detection is based on ECG processing as the methods listed above, one also finds proposals that incorporate different signals such as the photoplythysmogram (PPG). One example is the work of Solosenko et al., who dealt with the detection of ectopic beats by evaluating features derived from PPG recordings only. Two of their works target VEBs with an approach based on temporal features and variance [12] or power ratios [13] of the PPG signal after automatic artifact removal. Using ANN for the classification, they achieve a sensitivity and specificity above 92.4% and 99.9%, respectively. Another approach by Solosenko et al. proposes a set of simple, temporal and amplitude based features in combination with a Naive Bayes classifier to detect both VEBs and SVEBs [14]. The resulting performance of 96.4% sensitivity and 99.92% specificity is astonishing. The algorithms are evaluated on data drawn from the MIMIC and MIMIC II databases (Multiparameter Intelligent Monitoring in Intensive Care), as well as data obtained from clinical studies and with respect to unknown patients.

Drijkonigen et al. dealt with the transferability of ECG heartbeat interval features to PPG signals, recorded with a smartphone camera for ectopic beat discrimination [15]. By evaluating the peak to peak intervals within a single window, they successfully detected artifically induced supraventricular ectopic beats.

While it was demonstrated that ectopic beats can be detected from different signals, there has been little effort on multimodal approaches to the problem.

One work was proposed by Palaniappan et al. in 2004, who utilize a set of derivative based features from ECG and arterial blood pressure (ABP) signals, which are then presented to a backpropagation multi layer perceptron (MLP) for classification [16,17]. The not further specified classification performance of 96.47% on the MGH/MF database (Massachusetts General Hospital/Marquette Foundation) seems promising, although the evaluation scheme is not described in depth.

The novel beat detection algorithm proposed in this work adopts the general structure of the above presented approaches, including the steps of delineation, feature extraction and classification with the help of a properly trained architecture. In the first place, it was developed to support BPE applications, ideally yielding reasonable performance on different patients without prior per-patient training. Since PWV analysis applications have access to synchronized ECG and pulse wave signals anyway, the presented ectopic beat discrimination method derives features from both signals, similarly to the multimodal ABP-ECG method published by Palaniappan. As ABP signals are typically measured invasively and require trained medical staff as well as sterile conditions, the proposed PPG-ECG approach provides an interesting, non-invasive and non-occlusive alternative to extract pulse wave related parameters and timings, which is further suitable for ambulatory settings.

## 3. Materials and Methods

### 3.1. Pulse Wave Velocity Based on Electrocardiography and Photoplethysmography

Ambulatory electrocardiography derived by surface electrodes is a well developed concept, commonly applied with the help of small wearable Holter monitors. Recording the three ECG standard leads according to Einthoven already allows for an evaluation of the most important cardiac timings beginning from atrial depolarization (P-Wave) to ventricular contraction (R-Wave) and ventricular repolarization (T-Wave). Current literature provides a considerable inventory of automatic ECG processing algorithms, including wave delineation, artifact suppression or sophisticated feature extraction [18,19]. This has resulted in manifold practical applications such as heart rate variability monitoring, arrhythmia detection, analysis of myocardiac infarction, ischaemia or ECG derived respiration, just to mention a few important examples.

Photoplethysmography became popular in the early 1970s, when the first optical approach to non-invasively extract atrial oxygen saturation was introduced [20]. In recent years, the photoplethysmogram (PPG) was used to extract further physiological parameters including heart rate, respiratory activity, vascular analysis or cardiac rhythm assessment [21]. Technically, the PPG is acquired by light intensity measurements, which represent blood volume changes in the vascular bed. More detailed aspects are discussed in the following hardware section.

**Figure 1:**
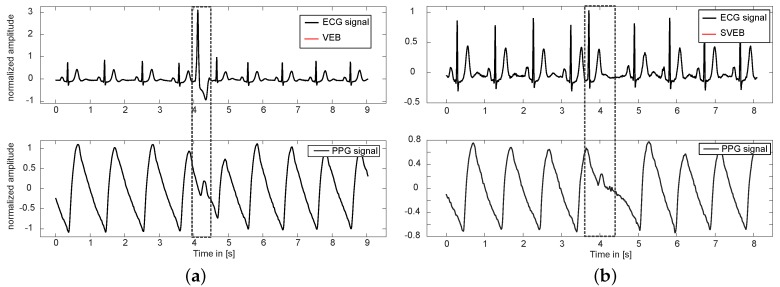
Synchronized electrocardiography (ECG) and photoplethysmography (PPG) waveforms. (**a**) single premature ventricular ectopic beat at t = 4 s; and (**b**) single premature supraventricular ectopic beat at t = 3.8 s. Both types of extrasystoles have a significant impact on the morphology of the arterial pulse wave, which degrades to a notch-like peak that is hard to distinguish from normal PPG dicrotic notches or slight motion artifacts. As a consequence, accurate timing considerations including pulse arrival time extraction are seriously hampered.

Combining ECG and PPG in synchronized measurements allows for the estimation of pulse wave velocity related parameters such as the pulse arrival time (PAT), which is often defined as the interval beginning at ventricular contraction until the arrival of the pulse wave in the peripheral arteries. Nonetheless, a robust beat to beat analysis of the pulse wave becomes infeasible when the morphology of single beats is affected by premature ectopic beats as depicted in Figure 1. Due to the early contraction, the left ventricular output volume is significantly decreased, resulting in a comparatively small premature pulse wave peak. Unfortunately, automatic delineation approaches, like the commonly applied peak detector according to Zong et al. [22], fail in reliably locating those ectopic pulse wave notches, which becomes even harder—if not impossible—in slightly noisy periods. These visual impressions confirm the concerns mentioned in the introduction and underline the need for ectopic beat detection, especially in unsupervised PAT/PTT applications.

### 3.2. Wearable Hardware System

Supporting multimodal signal processing in a wearable sensor network poses firm requirements on the hardware system. Wireless and unobtrusive devices are favored, especially when it comes to unsupervised long-time measurements. The measurements conducted in this work have been acquired using a Bluetooth synchronized body sensor network (BSN) whose technical details were published in [23]. This architecture provides a modular platform giving way to an easy integration of multiple sensors. In this work, the combination of a 12-channel ECG sensor and a transmission PPG module was employed to record the PWV relating signals. With respect to the ECG module, only the Einthoven lead II was considered, in order to extract R peaks and morphological ECG features. This ECG module is equipped with active electrodes, which provide an amplified analog ECG signal and also contain an acceleration sensor. Although motion issues are not further evaluated in the present context, this information was helpful for extracting periods of motion-artifact free signals.

**Figure 2:**
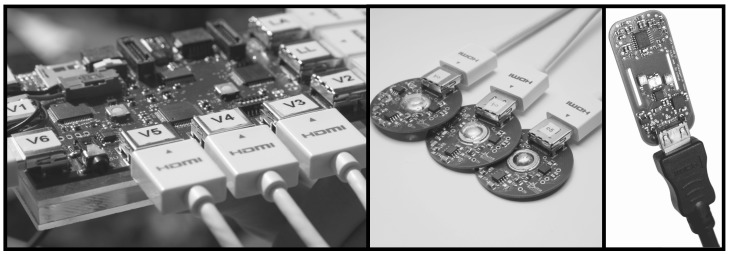
Assembled hardware system: (**a**) ECG sensor mainboard; (**b**) active ECG electrodes; and the (**c**) PPG module for acquiring the optical pulse wave signal.

The PPG sensor offers the acquisition of multi wavelength intensity signals, which permits more sophisticated applications such as atrial oxygen saturation extraction. In the present PWV recordings, only the infrared channel (λ=940 nm) was considered though. Figure 2 depicts the assembled hardware sensors of the deployed system. All signals are sampled at 500 Hz corresponding to a temporal resolution of 2 ms, which comes with a synchronization accuracy of 30 μs [23].

### 3.3. Multimodal Ectopic Beat Detection

The proposed ectopic beat detection method extracts a number of relatively simple features from preprocessed single-lead ECG and PPG signals. These features serve as inputs to a multilayer perceptron that discriminates normal and ectopic heartbeats. In order to calculate the features for one heartbeat, the preceding as well as the following ECG R-Peak and PPG pulse wave onset are considered. All R-Peaks are detected using the well known method proposed by Pan and Tompkins [24], whereas the pulse wave peaks are determined by a trivial maximum search between two adjacent R-Peak locations.

The implemented features are partly adopted from other works and extended by novel proposals, as is discussed next. A complete list of the features processed by the presented ectopic beat discriminator is given in Table 1, where every single feature can be derived by the R-Peak annotations and relies on trivial mathematical operations with fixed, deterministic execution time. Additionally, they are designed to be independent of the absolute amplitude of the input signals and do not need to be normalized with respect to other heartbeats. These properties allow for a simple and robust online implementation that only needs to buffer the signal between three ECG R-Peaks in order to calculate the features for one heartbeat. PPG and ECG features can be calculated separately on the corresponding sensor modules and are transferred to an arbitrary computation unit for the classification. In the proposed implementation, the classification application is hosted on one of the sensor nodes.

The first feature is inspired by the work of Chazal et al. [10], but, instead of calculating the preceding and following intervals of two consecutive R-Peaks (RR intervals) as separate features, the ratio of both is determined, which makes the feature independent of the current heart rate and sampling frequency. This feature has proven to reliably indicate SVEBs. The next three ECG features 2–4, which represent signal power, mean and max/min of the ECG signal amplitude aim at the distorted morphology resulting from VEBs. They are calculated for a window spanned by the starting and ending point of $ECG_{R}-0.35\times RR_{preceding}$ and $ECG_{R}+0.65\times RR_{following}$, respectively. Within the same window, the amplitude of the ECG signal is sampled at equally spaced points around the R-Peaks (features 5–14). These features, also inspired by Chazal et al.’s work [10], indicate abnormalities in beat morphology and have proven to benefit the detection of both classes of ectopic beats. The PPG fractional amplitude features 15–17, proposed by Teng et al. [25], capture the pulse wave morphology and are repurposed in this context. Features 18 and 19 are calculated in the same way as their ECG counterparts and represent the generally lower blood volume as a result of a premature contraction. They are calculated for an interval between two PPG onsets. Feature 20 relates the amplitude of the current pulse wave to the next one, which is often higher than a regular pulse wave following a normal heartbeat.

**Figure 3:**
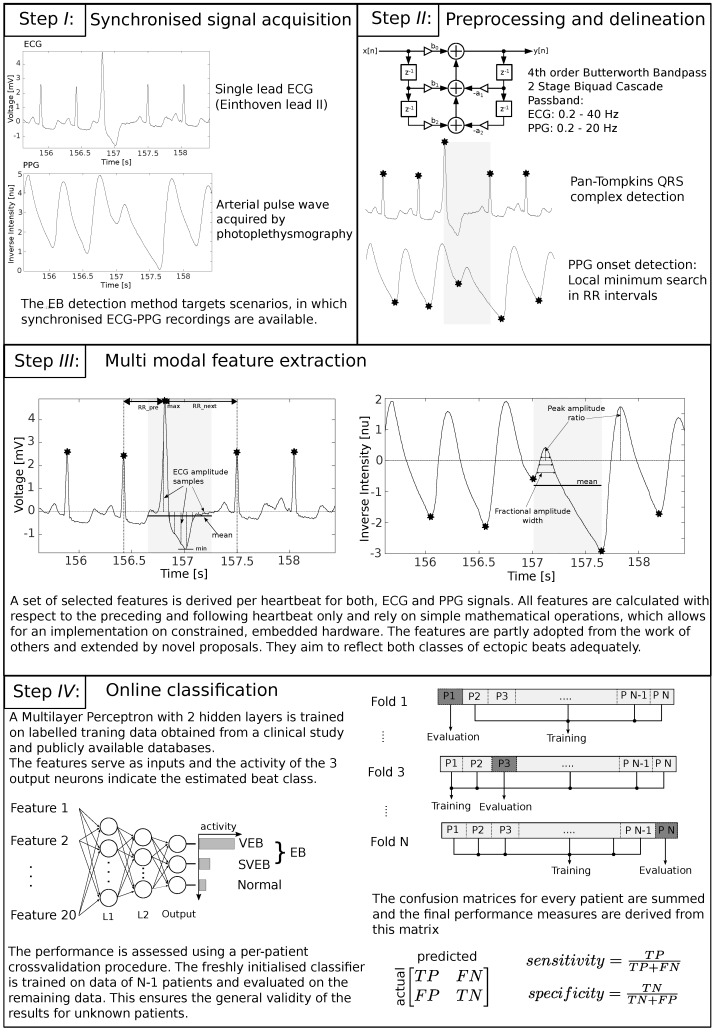
Overview on the proposed multimodal ectopic beat detection approach.

For the classification, a multilayer perceptron with 20 input neurons (features), two hidden layers with 10 and 8 neurons, respectively, and three output neurons (representing the heartbeat classes normal, SVEB and VEB) is randomly initialized during the training process. The number of hidden neurons is not critical; however, a tapering structure has shown to provide the best results. The training data is selected to represent all classes equally, i.e., to contain a similar number of the respective heart beat types. The network is trained using backpropagation along scaled gradient conjugate directions by presenting it with the features and the corresponding labels in the form of an incidence vector for all training data points. Early stopping is used to avoid overfitting. For this purpose, 10% of the datapoints in the training dataset are randomly chosen as a validation dataset and excluded from the actual training procedure. After each backpropagation step, the classification performance on the validation set is checked. As soon as this error increases with respect to the actual training error, the network is assumed to overfit the training data and training is stopped. When feeding a new feature vector to the input of the trained network, the output neuron with the highest output value represents the most probable class. The final goal is a binary classification of normal and ectopic beats; therefore, a beat is labeled as normal, if the corresponding neuron has the highest output, and as an ectopic beat in any other case. Figure 3 highlights the the most important steps of the whole approach again.

### 3.4. Blood Pressure Estimation

Having outlined the main aspects of ectopic beat detection, the second part of this work eventually concerns itself with the impact of extrasystoles during continuous blood pressure estimation. Therefore, this last subsection provides a short introduction to the basic backgrounds of BPE and introduces three different methodological approaches from published literature.

The concept of blood pressure estimation based on pulse wave velocity was already discussed in the late 1970s [26], mainly building upon the findings of Hughes et al., who investigated pressure–volume relations at arterial measurement sites in dogs and proposed an exponential description of the elastic modulus $E_{inc}$ as given in Equation (1):

(1) $$E_{inc}=E_{0}\times e^{\alpha P},$$

where *P* represents the corresponding atrial pressure and *α* is a constant of 0.017 mmHg${}^{-1}$ [27]. The Moens–Korteweg equation, as given in Equation (2), provides a direct link between the elastic modulus $E_{inc}$ and the pulse wave velocity $v_{pulse}$ of an incompressible fluid of density *ρ* in an elastic and cylindric vessel with wall thickness *h* and inner radius *r*:

(2) $$v_{pulse}=\sqrt{\frac{h\times E_{inc}}{2\times r\times \rho }}$$

As a matter of fact, the majority of works contributing solutions to solve the BPE problem are based on the above depicted Moens–Korteweg relation. Nonetheless, one also finds completely different approaches, which incorporate the evaluation of morphological pulse wave features. A changing pulse wave velocity is generally known to affect the shape of the pulse beat, as the reflected parts return earlier or later, thereby superimposing the primary waveform at a different location [28]. This notion might motivate a morphology based BPE implementation, associating blood pressure variations with PVW related changes of the underlying pulse shape.

The three chosen BPE methods were reimplemented in order to undergo a thorough performance analysis, especially in the presence of ectopic beats. It should be noted that the methods have been primarily selected according to their methodological approaches rather than their possible clinical significance, as the main goal was to demonstrate the invariable vulnerability of the single BPE solutions towards ectopic beats. Further potential influences including motion artifacts, hemodynamic conditions or postural changes are not incorporated into the following evaluation. Moreover, specific decisions pertaining to the implementation of the respective BPE methods (i.e., fiducial point choice in the PAT determination phase) were not questioned here, with the original specifications being straightly adopted as published by the corresponding authors. The key aspects of the corresponding approaches along with their original performance reports are discussed next.

The first method is the most cited work published by Chen et al. in 2000 [29], who—according to the Moens–Korteweg equation—proposed to derive a blood pressure estimate by evaluating changes in the pulse arrival time at discrete points in time *t*:

(3) $$P_{e}\left(t\right)=P_{b}-\frac{2}{\gamma T_{b}}\Delta T\left(t\right).$$

Here, $P_{b}$ refers to the last valid reference blood pressure measurement, serving as intermittent calibration. $T_{b}$ is the corresponding pulse arrival time of the last calibration, ΔT describes the measured change of PAT of the current beat and *γ* is a constant of 0.017 mmHg${}^{-1}$.

Chen et al. tested the performance on 20 patients during cardiovascular surgery, where the signals from chest lead ECG V5, a finger photoplethysmogram and the signal of an invasive blood pressure catheter were recorded. The performance was evaluated with the help of six different error measurements (cf. Table 2) including correlation coefficient (CC), root mean square error (RMSE), arithmetic mean of estimation errors as well as error distribution considerations.

Cattivelli et al. proposed a rather simple linear model, which is the second method considered in this work and incorporates PAT and heart rate (HR) measurements to derive a pressure estimate for systole and diastole [30]:

(4a) $$SBP=a_{1}\times PAT+b_{1}\times HR+c_{1},$$

(4b) $$DBP=a_{2}\times PAT+b_{2}\times HR+c_{2}.$$

This model is initially fitted by means of multiple calibration measures ($N_{cal}$ = 40) and is intermittently recalibrated using a recursive least squares algorithm. The performance was assessed on records drawn from the MIMIC database by evaluating the three error measurements: mean error, standard deviation (SD) of error and mean squared error (MSE) as summarized in Table 3.

In addition to the two above presented PAT based approaches, a completely different technique incorporating morphological pulse wave features is examined as well.

Kuryliak et al. proposed a BPE approach, which relies on PPG waveform features only, and chose a feed-forward multi-layer artificial neural network to solve the SBP and DBP estimation tasks [31]. In total, they analyzed 15,000 beats drawn from the MIMIC database, where 70% were used for training, resulting in a quite passable performance with mean SBP errors of 3.8±3.46 mmHg and mean DBP errors of 2.21±2.09 mmHg (although it was not explicitly stated that different datasets of a single patient are exclusively used for either training or testing). The neural network was fed with 21 features exclusively describing the morphological shape of the pulse wave such as systolic and diastolic widths at different heights (10%–70%) of the pulse peak amplitude. These features have been studied in previous pulse wave analysis applications, showing that they contain useful physiological information [25,32].

Concerning the results, Kuryalak adopted two different performance measures (cf. Table 4) including the absolute error of the estimated blood pressure and a relative error that is defined as the absolute error divided by the corresponding reference blood pressure value.

**Figure 4:**
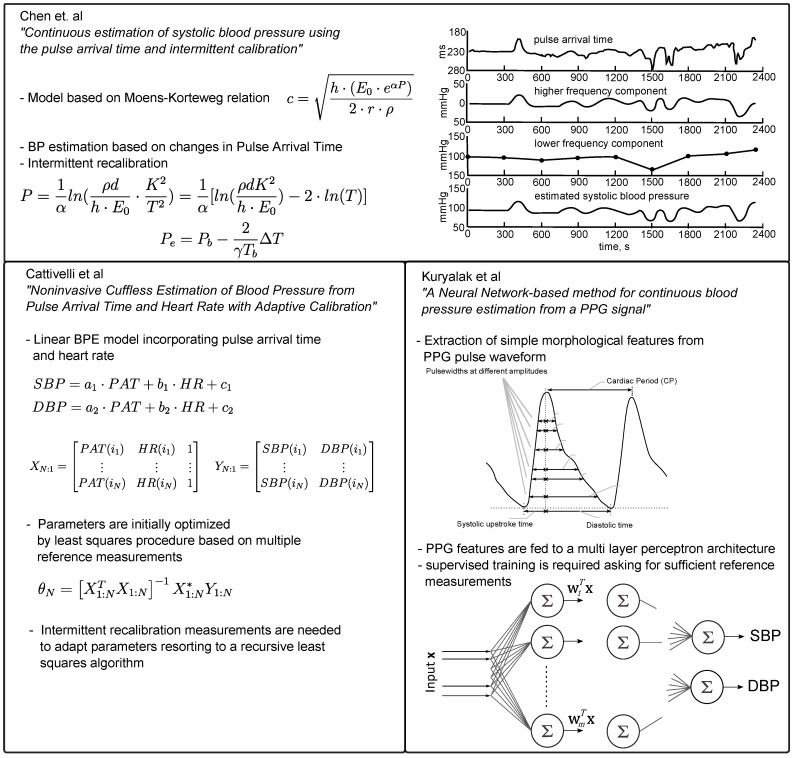
Overview on the implemented blood pressure estimation methods. Figures have been redrawn from the respective publications [29,30,31].

A complete overview of the presented approaches is given in Figure 4, which summarizes the implementational details of the respective methods.

At this point, it is noted that this work does not cover a comprehensive evaluation of the respective BPE methods themselves. The underlying processes and evaluation schemes are not further scrutinized, as the current study will only concentrate on the influence of ectopic beats on the BPE procedure.

### 3.5. Data Acquisition/Databases

To investigate the applicability in clinical and ambulatory environments, the ectopic beat algorithm is exercised on data recorded with the previously introduced ECG and PPG hardware. Therefore, a small clinical study was prepared, including patients of the geriatric units from ”Städtisches Klinikum Dresden, Germany” and ”HELIOS Klinikum Aue, Germany” hospitals, who were selected by a cardiologist with respect to the occurrence of ectopic beats. The study was confirmed by the ethical board at Technical University of Berlin (request PF_01_20140513), and all participants declared their voluntariness and consent.

As an addition to the records gained from these measurements, another source of data was required for a larger amount of training data and better significance of the evaluation results. The Physionet Challenge 2015 (PC15) training database contains 750 datasets recorded from different patients in clinical environments [33], which are publicly available on the Physiobank database [34]. Inclusion criteria asked for the existence of ECG lead II and a PPG channel, which are required to derive the features of the presented algorithm. Moreover, a sufficient presence of ectopic beats was demanded, whereas records and segments with heavy artifacts in any of the channels were excluded, resulting in a total of 31 datasets.

The synchronicity among the signals in the PC15 records occasionally appeared to be untimely, which became obvious when matching the ECG R-Peak to the corresponding PPG pulse wave. In some cases, a large offset between the ECG and PPG recordings was recognized, which was probably introduced by the original measurement setup. In the current processing, this offset is compensated by shifting the signals to fulfill the aforementioned assumption that the resulting pulse wave in the PPG signal follows the R peak of the corresponding heartbeat in the ECG signal. With regards to PWV applications, the affected records are completely useless, but they can still be used to evaluate the proposed EB detection algorithm without any concerns.

For training as well as evaluation, labeled datasets are required. The correctness of these labels is a delicate factor, as it directly affects the overall classification performance. Therefore, all records were first annotated by two non-experts, followed by a thorough review by an expert cardiologist. Segments around ambiguous heartbeats were excluded. The resulting database, used for evaluation of the proposed ectopic beat detection method, contains data of 35 patients. The individual records and corresponding number of annotated beats are listed in Table 5.

In order to evaluate the introduced blood pressure estimation approaches, further records providing synchronized ECG, PPG and ABP channels are required. The MIMIC database is one of the biggest available collections of intensive care unit records, which is also distributed by PhysioNet. In the present work, records from the first volume [35] were extracted, which provides both episodes with and episodes without occurring ectopic beats. Moreover, two of the considered BPE methods were evaluated on data drawn from this database in the corresponding original publications.

## 4. Results

### 4.1. Ectopic Beat Detection Performance

A per-patient cross validation procedure is adopted to evaluate how well the detection algorithm performs. A new instance of the network is trained on the data from N−1 patients, where n=35 equals the total number of patients in the database as listed in Table 5. The trained network is then presented with the features of the remaining data of one patient and the resulting output is compared to the ground truth to calculate a confusion matrix. This procedure is repeated for all *N* datasets, where the confusion matrices are summed up in each step. The final performance measures—sensitivity and specificity—are derived from that matrix, describing the whole database.

Sensitivity is defined as the ratio of the number of correctly labeled ectopic beats to the total number of beats labeled as ectopic. Specificity is the ratio of the number of correctly labeled normal beats to the total number of beats labeled as normal. In the context of ectopic beat removal for blood pressure estimation, a high sensitivity lowers the influence of ectopic beats on the detection algorithm. The specificity on the other hand is not inherently crucial regarding the performance. However, a low specificity limits the number of sampling points, which, in turn, affects the achievable accuracy. The applied evaluation scheme guarantees that the resulting performance is generally valid for unknown patients, since the classifier is never trained on data obtained from the same patient as it is evaluated for.

To examine the usefulness of the multimodal approach, the evaluation is repeated for features from both signals separately (Features 1–14 and Features 15–20, see Table 6). The input layer of the MLP is changed accordingly to reflect the reduced number of features. Additionally, the per class sensitivity is calculated in order to verify whether the proposed solution is able to detect both VEBs and SVEBs.

Moreover, the complete algorithm has been implemented in the earlier depicted hardware system. The main controller was upgraded to an TI MSP432 MCU (Texas Instruments, Dallas, TX, USA), which is based on a 32-Bit ARM Cortex-M4 (ARM Holdings, Cambridge, UK) and has access to an integrated floating point unit. The filter and delineation routines work on blocks of 64 samples. A complete heart beat processing step is then executed in less than 300,000 CPU clock cycles and approximately requires 4.5 kBytes RAM. More details are found in Table 7.

### 4.2. Blood Pressure Estimation Performance

In the interest of elaborate performance tests, all three BPE methods were reimplemented in a MATLAB (MathWorks, Natick, MA, USA) environment, based on the information given in the respective publications. As indicated earlier, the performance was assessed on selected records of the MIMIC database, which provide extracts with and without ectopic beat presence. In total, three setups have been considered. First, every BPE implementation was run on clean signals without any extrasystoles to compare the outputs with the originally published result reports. Next, the BPE routines are exercised on datasets containing a significant amount of ectopic beats, which is assumed to have a serious impact on the performance of all BPE processes. In the last and third run, the same signals from the second step are presented to the algorithms again, this time preprocessed by the proposed EB discriminator, removing extrasystoles of any kind for the subsequent BPE procedure. The corresponding results are summarized in Table 8, listing the different performance measures that were discussed in the previous sections.

**Figure 5:**
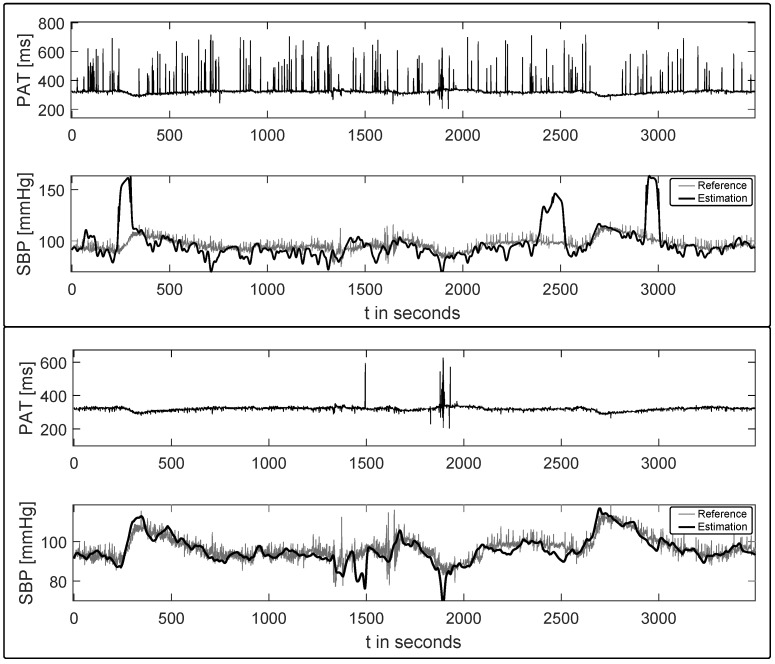
Example Blood pressure estimation output (Chen’s method [29]) before and after automatic ectopic beat clearance. The pulse arrival time (PAT) trace is given in the top plots, whereas the continuous systolic blood pressure (SBP) reference along with the estimated SBP (bold) are plotted at the bottom.

Figure 5 gives a visual impression on the influence of EBs on an example BPE process (based on Chen’s approach [29]). The upper part presents two timelines where the PAT signal is drawn on the top and the estimated systolic blood pressure (bold) along with the continuous SBP reference in the bottom plot. The ectopic beats can be identified by the sharp deflections in the PAT trace, which are due to the difficulties in detecting the pulse wave onsets (cf. discussion in Section 3.1). The overall estimation output is clearly hampered, with even more severe deviations occurring when a recalibration routine is based on an inaccurate PAT value caused by an extrasystole. The second plot, on the other hand, presents the same signal extract, where all PAT values that were determined during an underlying ectopic beat have been discarded, resulting in a roughly undisturbed PAT signal.

## 5. Discussion

The results in Table 6 demonstrate the effectiveness of the proposed EB detector. Unfortunately, a comparative analysis with respect to the published methods introduced earlier is hard to realize, due to the lack of a common, annotated reference database and consistent performance measures. Moreover, an impeccable comparison would require accurate reimplementations of the original works, which could not be provided in this work. Nonetheless, the achieved performance values of the proposed EB detector in terms of sensitivity and specificity are favorable, especially under the constraint that training and testing was performed on records of different patients. The presented method is able to reliably detect and distinguish both classes of ectopic beats and could therefore be extended to a heartbeat classifier for other application scenarios. As shown in the second row of Table 6, the classification based on the implemented ECG features performs well already. This finding is consistent with the popular focus on ECG signals for detection of ectopic beats. In contrast, the PPG only detection (Table 6, row 1) seems to underperform, especially for SVEBs. The high performance achieved by other published PPG based methods might therefore call for a revision of the implemented PPG features. The improved sensitivity of the multimodal approach, however, underlines the benefit of combining features from both signals. Moreover, it was successfully demonstrated that all stages of the algorithm can be executed on state-of-the-art low power microcontrollers, which is a significant advantage of the proposed EB detector.

The second aim of the current analysis concentrated on the effects of ectopic beats on the accuracy of different BPE procedures. As mentioned in the introduction, this issue has given rise to particular concern in recent publications [5] and requires reliable solutions when BPE methods are to be applied in practical and unsupervised settings. The basic performance of the respective BPE reimplementations was examined in advance, to assure a proper functioning of the respective methods before they were applied for a solid evaluation regarding EB influences. As shown in Table 8a, the test runs of the three BPE approaches yield SBP estimation results comparable to the values reported in the original publications, indicating correctness of the corresponding reimplementations. It is stressed that the three methods have been chosen due to their different nature of approaching the BPE process in order to check whether all models are affected by ectopic beat presence to the same extent. The absolute values of the performance measures are not further scrutinized here, as this work only concentrates on the relative changes before and after ectopic beat clearance. A thorough interpretation of these values would actually require much more effort and especially more detailed information on the single datasets. Drawing conclusions from a selected error measurement like the MSE, for example, only makes sense if the overall properties of the record under testing are known. Datasets containing segments with significantly varying PAT and BP values pose a clearly higher demand on the BPE process when compared to extracts consisting of steady and nearly constant signals. Unfortunately, such considerations are neglected in most of the original publications, calling for future efforts and comparative investigations.

When analyzing the BPE performance during periods of ectopic beats, as is presented in Table 8b, one clearly recognizes the notable drops of the single error measures. The estimation outputs partly exceed physiological meaningful boundaries, underlining the serious difficulties in handling a reliable pulse wave timing extraction during ectopic beats.

Table 8c demonstrates the capability of the proposed multimodal ectopic beat discriminator to remove the previous encountered outlier when it is applied for EB clearance prior to the BPE process, resulting in feasible performance values again. It should be kept in mind, however, that for each detected ectopic beat, the corresponding pulse wave feature and its connected BPE sample is discarded. Thus, a continuous blood pressure estimation during periods of ventricular tachycardia, for example, would not be possible anymore.

## 6. Conclusions

This work contributed an effective ectopic beat discrimination algorithm applied for online cancellation of extrasystoles in blood pressure estimation applications. It was shown that the inclusion of multimodal features significantly improves the sensitivity and specificity of the ectopic beat detection process, which is interesting for applications that have access to synchronized PWV signals anyway. The low computational complexity of the proposed algorithm is one of the major advantages, which allows implementations on battery driven sensor nodes, whose possibilities are typically restricted due to the limited hardware resources.

In order to analyse the impact of EB on BPE methods, three individual proposals from recent literature were reimplemented and tested on different datasets. The estimation performance was assessed before and after ectopic beat discrimination resorting to the novel multimodal EB detector. It was generally shown that the presence of ectopic beats poses a potentially underestimated source of errors, introducing BPE inaccuracies, which make unsupervised applications very difficult if not impossible.

Applying a reliable ectopic beat detector prior to any BPE procedure is therefore mandatory and should be incorporated in any BPE setups. The proposed multimodal EB discriminator does not only provide a well performing architecture to support current works in the promising field of continuous and non-invasive blood pressure estimation, but can also contribute a positive impact on related applications including biomarker extraction from intraarterial pressure or pulse tonometry curves.

With regards to a comparative performance analysis of the BPE methods themselves, additional aspects including changing hemodynamic situations, motion artifacts, frequency of calibration routines, postural changes and further environmental factors still need to be considered. This leaves open questions for future works on the way to assess the feasibility of BPE approaches in practical situations.

## Figures and Tables

**Table 1:** Electrocardiography (ECG) and photoplethysmography (PPG) derived features, which are extracted from each heart beat and processed by the presented multimodal ectopic beat detector. All features can be calculated with information drawn from the preceding and following heartbeat only, which allows for a small memory footprint and low detection delay. Furthermore, they are designed to adequately reflect both major classes of ectopic beats.

Number	Description	References
1	leading/trailing interval of consecutive R-Peaks (RR)	[10]
2	ECG heartbeat power	
3	ECG hearbeat mean	
4	ECG heartbeat max/min	
5–14	samples around R peak	[10]
15–17	PPG fractional amplitude	[25]
18	PPG pulse wave power	
19	PPG pulse wave mean	
20	current/next PPG pulse peak amplitude	

**Table 2:** Blood pressure estimation approach after Chen et al. [29]: Performance measures applied in the original publication including mean error, correlation coefficient, root mean square error (RMSE) and a probability of error distribution (Prob. 10% = range of normalized error within 10%)

Mean Error	RMSE	CC	Prob. 0%	Prob. 10%	Prob. 16%
0.06 mmHg	3.70 mmHg	0.97	38.8%	97.8%	99.4%

**Table 3:** Blood pressure estimation (BPE) approach after Cattivelli et al. [30]: performance measures applied in the original publication evaluating the systolic blood pressure (SBP) estimation routine. Diastolic blood pressure (DBP) estimation is not considered in this work.

Mean Error	Standard Deviation of Error	MSE
−0.41 mmHg	7.77 mmHg	70.05 mmHg${}^{2}$

**Table 4:** Blood pressure estimation (BPE) approach according to Kuryalak et al. [31]: performance measures applied in the original publication. Absolute error: absolute magnitude between estimated systolic blood pressure (SBP) and reference SBP; relative error: absolute error divided by corresponding reference SBP.

Absolute Error	Relative Error
3.80±3.46 mmHg	3.48%±3.19%

**Table 5:** Records used for evaluation of the ectopic beat detection algorithm. The database consists of data recorded as part of a small study using the referenced body sensor network as well as records drawn from the PhysioNet Challenge 2015 database [33].

Database	Record	# N	# Ventricular Ectopic Beats	# Supraventricular Ectopic Beats
rBSN	au_03	1381	4	27
rBSN	dd_02	645	0	70
rBSN	dd_03	141	6	7
rBSN	dd_06	688	4	3
PC15	a624s	302	1	2
PC15	a746s	406	5	0
PC15	b340s	254	0	22
PC15	b341l	260	0	23
PC15	b515l	211	2	4
PC15	b517l	227	0	6
PC15	b560s	139	10	17
PC15	b562s	123	29	11
PC15	b838s	242	20	28
PC15	f642s	457	0	8
PC15	t416s	240	1	40
PC15	t662s	567	6	0
PC15	t680s	348	15	3
PC15	t752s	383	0	5
PC15	t762s	313	5	38
PC15	v132s	227	15	0
PC15	v158s	77	6	1
PC15	v205l	87	10	9
PC15	v253l	535	77	0
PC15	v254s	441	35	3
PC15	v255l	445	47	0
PC15	v368s	329	6	0
PC15	v427l	175	0	17
PC15	v557l	264	4	0
PC15	v559l	354	23	9
PC15	v573l	335	0	2
PC15	v648s	340	2	1
PC15	v696s	237	0	46
PC15	v769l	357	25	1
PC15	v831l	319	15	0
PC15	v833l	217	13	3
TOTAL		12066	386	406

**Table 6:** The evaluation is conducted on the complete set of features and also on ECG and PPG features separately. The sensitivity is calculated for all ectopic beats and for both classes, respectively.

Set of Features	Sensitivity	Sensitivity SVEB	Sensitivity VEB	Specificity
PPG	77.7	68.2	87.6	95.5
ECG	91.12	87.2	95.3	98.9
All	95.7	96.1	95.3	99.0

**Table 7:** The EB detection algorithm is implemented on the TI MSP432 MCU. Clock cycles and RAM usage are measured for every step of the algorithm. The results are given for a sample rate of 500 Hz and a heart rate of 72 beats per minute (bpm). For the calculation of the total values, the distributed nature of the algorithm is taken into account.

		Clock Cycles		RAM Usage (Byte)	
Step	per	ECG	PPG	ECG	PPG
Filter	64 samples	2414	2414	68	68
Delineation	sample	418	75	388	44
Feature Extraction	heartbeat	8822	8445	4000	4000
Classification	heartbeat	980		1200	
Total	heartbeat	283,239		4456	

**Table 8:** Blood pressure estimation (BPE) performance evaluation. (**a**) BPE error measurements on clean datasets to verify the respective BPE reimplementations; (**b**) BPE error measurements on datasets containing ectopic beats; (**c**) BPE error measurements on the same datasets used in (**b**) where the ectopic beat cancellation using the multimodal method proposed in this work has been applied prior to the BPE process.

Method	Mean Error	SD Error	CC	MSE	RMS	$E_{A}$	$E_{R}$
(**a**) BPE performance measures on datasets with no ectopic beats							
Chen	0.26	3.41	0.84	11.71	3.42	2.46±2.38	2.55±2.52
Cattiveli	0.13	5.22	0.74	27.24	5.22	2.126±4.77	2.22±5.21
Kuryalak	−0.0855	3.87	0.77	14.81	3.85	2.96±2.46	3.89±2.67
(**b**) BPE performance measures on datasets with ectopic beat presence							
Chen	0.96	9.41	0.2966	89.36	9.45	6.98±6.37	7.15±6.63
Cattiveli	4.2465	25.6	0.07	674.5	25.97	6.32±25.19	6.32±24.71
Kuryalak	−83.9	8.01	0.13	7112	84.3	83.9±8.1	87.4±12.04
(**c**) BPE performance measures on datasets with ectopic beat presence, after prior EB clearance							
Chen	−0.81	5.09	0.88	26.7	5.17	3.81±3.51	4.02±3.67
Cattiveli	0.17	4.23	0.91	17.9	4.24	2.92±3.07	3.06±3.14
Kuryalak	0.05	4.41	0.84	19.41	4.41	3.29±2.93	3.55±3.26

## Abbreviations

The following abbreviations are used in this manuscript:

ABP: Arterial Blood Pressure
ANN: Artificial Neural Network
BPE: Blood Pressure Estimation
CC: Correlation Coefficient
DBP: Diastolic Blood Pressure
EB: Ectopic beat
ECG: Electrocardiography/Electrocardiogram
HR: Heart Rate
HRV: Heart Rate Variability
ICA: Independent Component Analysis
LDA: Linear Discrimination Analysis
PCA: Principal Component Analysis
PPG: Photoplethysmography/Photoplethysmogram
MGH/MF: Massachusetts General Hospital/Marquette Foundation
MIMIC: Multiparameter Intelligent Monitoring in Intensive Care
MIT-BIH: Massachusetts Institute of Technology - Beth Israel Hospital
MLP: Multi Layer Perceptron
MSE: Mean Squared Error
PAT: Pulse Arrival Time
PNN: Probabilistic Neural Networks
PTT: Pulse Transit Time
PWV: Pulse Wave Velocity
RMSE: Root Mean Square Error
RR: interval of two consecutive ECG R-Peaks
SBP: Systolic Blood Pressure
SD: Standard Deviation
SVEB: Supraventricular Ectopic Beat
SVM: Support Vector Machine
VEB: Ventricular Ectopic Beat
